# A new species of *Microlicia* (Melastomataceae): first record of the genus for Colombia

**DOI:** 10.3897/phytokeys.122.34171

**Published:** 2019-05-28

**Authors:** Humberto Mendoza-Cifuentes, William Ariza, David E. Granados, Rosana Romero

**Affiliations:** 1 Instituto Alexander von Humboldt, Herbario FMB, Carrera 8 # 15-08, Claustro de San Agustín, Villa de Leyva, Colombia Instituto Alexander von Humboldt Villa de Leyva Colombia; 2 Universidad Distrital Francisco José de Caldas, Herbario Forestal UDBC. Carrera 3 # 26A-40, Bogotá, Colombia Universidad Distrital Francisco José de Caldas Bogotá Colombia; 3 Investigador Independiente. Bogotá, Colombia Unaffiliated Bogotá Colombia; 4 Universidade Federal de Uberlândia, Instituto de Biologia, Av. Amazonas, 20 – Umuarama, Uberlândia – MG, 38405-302, Minas Gerais, Brazil Universidade Federal de Uberlândia Minas Gerais Brazil

**Keywords:** Northeastern Andes, endemism, long-distance seed dispersal, northern Andes, sub-páramo

## Abstract

*Microlicia* (Melastomataceae) is a Neotropical genus nearly restricted to southeastern Brazil, and the Guiana Shield in Venezuela, with a few species in some places in the Andes of Bolivia and Peru. A new species of *Microlicia* endemic to the mountains of eastern Andes of Colombia is described and illustrated. Its affinities with other morphologically similar species from Venezuela are also documented. This novelty is the first record of the genus for Colombia and the northern Andes. It is argued that this disjunct distribution of the genus is attributable to the phenomenon of long-distance seed dispersal by wind.

## Introduction

*Microlicia* D. Don is a genus of approximately 140 species, 135 of which occur in Brazil, two species grow in the Guaiana Shield of Venezuela, and two in the Andean mountains of Peru and Bolivia (Michelangeli and Cotton 2008, Romero 2013, Romero and Woodgyer 2015, Flora do Brasil 2019). The genus is extremely diverse in the campos rupestres and savannah vegetation of the states of Bahia, Minas Gerais and Goiás in Brazil (Romero 2003a, 2013).

The species of *Microlicia* are generally characterized by solitary flowers with five or occasionally six petals, a superior ovary with three or occasionally five locules, and capsules dehiscing longitudinally from the apex to the base (Almeda and Martins 2001, Romero 2003b). Difficulty in the delimitation of closely related species may explain the high number of names proposed for several species complexes in the genus (Romero and Woodgyer 2014).

This novelty represents the first record of the genus for Colombia and the northeastern Andes. The area from which this new taxon comes is poorly collected due to the difficult access and social conflict in the region. The current peace process in Colombia and the project Boyacá BIO developed by the Alexander von Humboldt Institute and the Government of the Department of Boyacá has allowed access to these new biologically unexplored areas.

## Material and methods

This new species was found in the course of reviewing collections in regional herbaria of Colombia generated by the Boyacá BIO project. After several collections were located in the Federico Medem (FMB, Villa de Leyva) and Universidad Distrital (UDBC, Bogotá) herbaria, it was possible to visit the localities and collect new samples. Therefore new collections, field photographs and samples of flowers, fruits, and seeds stored in 70% alcohol were made.

Measurements of vegetative parts were made in the dry herbarium material with a digital caliper with a precision of 0.1 mm. The measurements of floral parts were based on fresh flowers preserved in alcohol from the type specimens. A Leica S8AP0 microscope was used. Photographs of leaves, flowers, fruits, and seeds were taken in the field and laboratory from fresh material using an MC190 HD camera.

The distribution of the *Microlocia* was associated with South America wind currents to evaluate the possible cause of the presence of the new taxon in the Andes of Colombia. For this, the map of winds patterns from the Earth Nullschool (https://earth.nullschool.net) was used, on which the distribution records of *Microlicia* from the electronic Tropicos database of the Missouri Botanical Garden (http://www.tropicos.org) were mapped using Arc-GIS version 10.2.1.

## Results

### Taxonomic treatment

#### Microlicia colombiana

Taxon classificationPlantaeMyrtalesMelastomataceae

HumbertoMend. & R.Romero sp. nov.

urn:lsid:ipni.org:names:60478838-2

Figs 1 , 2 , 3

##### Diagnosis.

Related to *Microliciabenthamiana* Triana but differs in having larger internodes, strigose nodes, linear to obovate leaf blades, trichomes between sepals, petals and pedoconective of antesepalous stamens shorter. Also related to *Microliciaguanayana* Wurdack but differs in having non-strigose adaxial foliar surfaces, 3-nerved leaf blades, hypanthia 10-ribbed, petals < 8.5 mm long and with a rounded setose apex, and stamens with smaller dimensions.

TYPE: COLOMBIA. Boyacá: Municipio de Pisba, vereda Miraflores, Sabana de Nubacá, 2389 m elev., 5°44'7.10"N, 72°37'32.02"W, 19 Nov 2018 (fl, fr), *H. Mendoza & D. Granados* 22014 (holotype: FMB!; isotypes: COL!, CUVC!, HUA!, FMB!, CUVC!, UDBC!).

##### Description.

Denselly branched shrub 70–190 cm tall; internodes 2.4–8.1 mm long, 0.3–1.1 mm wide; young branches quadrangular becoming terete in old-basal parts, glabrous or puberulous with long-stalked glands 0.3–0.6 mm long, the stalk is curled, glands early caducous; nodes strigose, trichomes similar to the ones in the internodes; distal internodes with sessile glands. Leaves decussate, isophyllous, ascending, adaxial surface green (fresh material), abaxial surface clear pale green (fresh material), subsessile; petiole 0.3–0.7 mm long and 0.2–0.5 mm wide; blade linear to oblong, 4.1–9.9 × 1.1–2 mm, apex rounded, mucronate, frequently with a terminal seta 0.2–0.4 mm long, margin entire, revolute, frequently with sparse trichomes ca. 0.3 mm long, adaxial surface with spherical, golden glands, 0.03–0.06 mm diameter, abaxial surface glabrous or impressed with spherical golden glands 0.03–0.04 mm diameter, central vein with pale trichomes 0.2–0.3 mm long; 3-nerved or slightly plienerved to 0.4 mm from the base (visible only on adaxial surface), middle vein impressed on adaxial surface. Flowers 5-merous, axillary, among the distal leaves, solitary, diplostemonous; pedicel 1.2–2 mm long. Hypanthium 3–3.7 × 2.2–2.5 mm, obconical or terete, externally 10–ribbed, with short-stalked glands, glands 0.03–0.04 mm diameter; internally glabrous. Calyx lobed; undivided part of 0.2–0.3 mm long; lobes 2.1–2.7 × 0.9–1.4 mm (excluding seta), narrowly triangular, apex acute with a terminal seta 0.3–0.9 mm long, adaxial surface glabrous, abaxial surface towards the base with short-stalked glands similar to the hypanthium; pale trichomes 0.6–1 mm long intercalated with lobes. Petals 6.8–8.5 × 4.3–5 mm, obovate, pink, darker in the venation, glabrous, apex rounded. Stamens 10, strongly dimorphic, glabrous, anthers polysporangiate; small (antepetalous) stamens 5, filaments 2.6–5.3 × 0.2–0.4 mm, glabrous, light purple; pedoconnective 0.7–1.5 mm long, arcuate, yellow; ventral appendage 0.3–0.7 × 0.3–0.4 mm, globose or lobed, yellow; thecae 1.8–2 × 0.5–0.8 mm (including the rostrum), oblong, yellow, rostrum 0.3–0.4 mm long, with a ventrally oriented pore 0.1–0.3 mm diameter; large (antesepalous) stamens 5, filaments 3.2–4.9 × 0.2–0,4 mm, glabrous, light purple; pedoconnective 1.9–2.9 mm, ventrally arcuate, light purple; ventral appendage 1.1–2.2 × 0.9–1.1 mm, spatulate, light purple towards the base and yellow towards the apex; thecae 2.2–2.4 × 0.6–0.7 mm (including the rostrum), oblong, red, rostrum 0.3–0,5 mm long, with a ventrally oriented pore 0.1–0.2 mm diameter. Ovary 1.6–2.5 × 0.8–1.4 mm, oblate, superior, 3-locular, glabrous, apex rounded; style 3.3–5.2 mm long; stigma punctiform, 0.1–0.2 mm diameter. Capsule 3.2–3.4 × 2.1–2.3 mm, oblong, dehiscing into 3 valves from the apex, enveloping hypanthium early caducous. Seeds ca. 0.6 × 0.2–0.3 mm, ovoid to oblate, beige; testa lightly reticulate.

**Figure 1.**
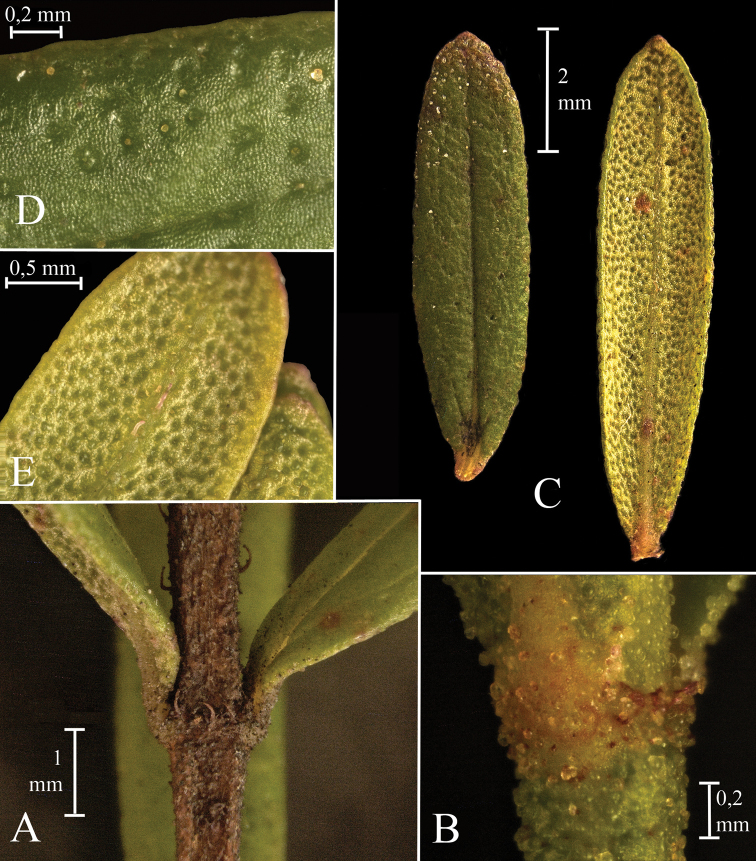
*Microliciacolombiana* HumbertoMendR.Romero. **A** Node at basal portion of a branch **B** Node with spherical golden glands at the distal part of a branch **C** Leaves, adaxial (left) and abaxial (right) surfaces **D** Leaf adaxial surface, with detail of indumentum **E** Leaf abaxial surface, with detail of indumentum (**A–E** from *H. Mendoza & D. Granados 22014* (FMB). All photos by Humberto Mendoza-Cifuentes).

**Figure 2.**
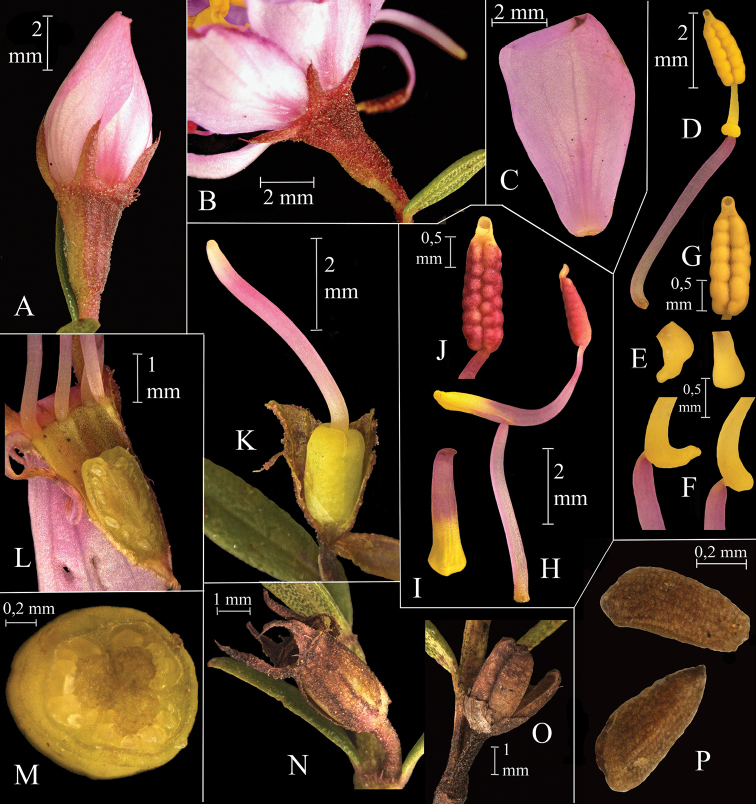
*Microliciacolombiana* HumbertoMend.&R.Romero. **A** Floral bud **B** Lateral view of the calyx and hypanthium in open flower **C** Petal **D** Antepetalous stamen **E** Top view of ventral appendage of the connective **F** Lateral view of ventral appendage of the connective **G** Polysporangiate anther **H** Antesepalous stamen **I** Top view of ventral appendage of the connective **J** Anther **K** Gynoecium **L** Longitudinal section of the ovary **M** Horizontal section of the ovary **N** Capsule covered by hypanthium **O** Open capsule **P** Seeds (A–P from H. *Mendoza & D. Granados 22014* (FMB). All photos by Humberto Mendoza-Cifuentes).

##### Phenology.

Collected with flowers and fruits in July and November. In the area, there is only one rainy period with the highest levels of precipitation between the months of May to July and with less precipitation between December to February (Jaramillo and Chaves 2000). Flowering coincides with less rainy periods.

##### Habitat and distribution.

**Figure 3.**
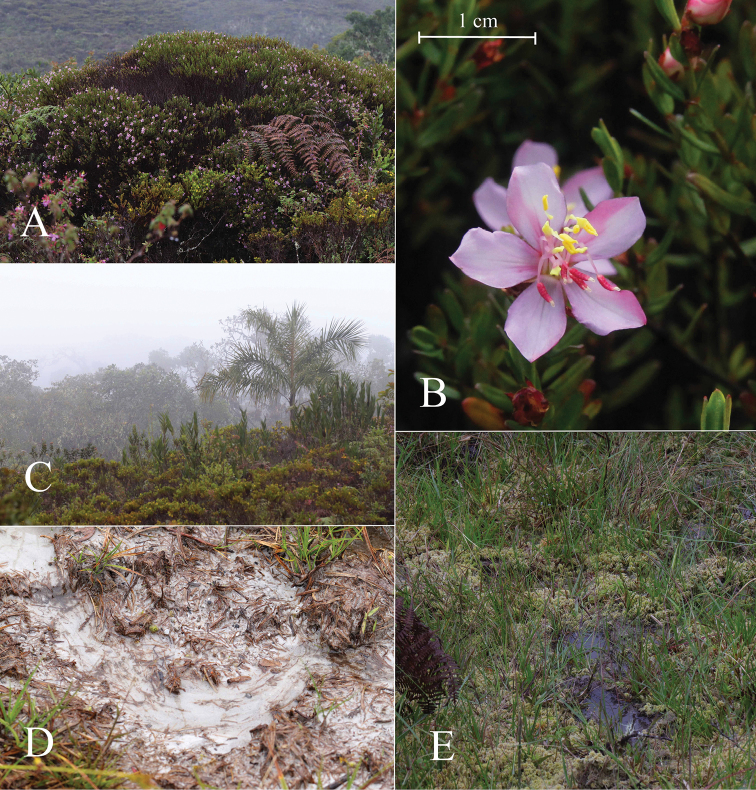
*Microliciacolombiana* HumbertoMend.&R.Romero. **A** Habit **B** Flowering branch **C** Sub-páramo habitat at te type locality **D, E** Sandy soils and substrate of the type lovality (all photos by David Granados).

**Figure 4.**
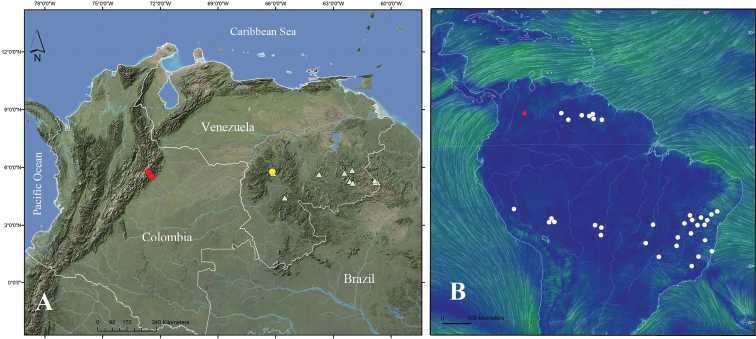
Distribution of *Microlicia*. **A** Distribution of *M.colombiana* and the most similar species in northern South America (Red dots: *M.colombiana*; Yellow point: *M.guanayana*; Green triangles: *M.benthamiana*) **B** Distribution of *Microlicia* in South America and wind currents (Red point depicts distribution of *M.colombiana*, white dots are other species of the genus).

*Microliciacolombiana* is endemic to northeastern Andes of Colombia towards the Orinoquian flank. This species is only known from the type locality in the department of Boyacá near the southern border of Pisba National Natural Park, and a nearby second locality in the department of Casanare, between 2000 to 2400 m elevation (Figure 4A). It grows in open areas of sub-páramo vegetation on white sandy soil covered with sphagnum (Figure 3).

##### Etymology.

The specific epithet refers to the restricted occurrence of the new species to Colombia and for being the first record of the genus in this country.

##### Specimens examined

**(Paratypes). COLOMBIA. Boyacá**: Municipio de Pisba, vereda Miraflores, Sabana de Nubacá, 2389 m elev., 5°44'7.10"N, 72°37'32.02"W, 1 nov 2017, fl, fr, *D. Granados 811* (CUVC, FMB); vereda Miraflores, Sabana de Nubacá, 2389 m elev., 5°44'7.10"N, 72°37'32.02"W, 19 nov 2018, fl, fr, *H. Mendoza & D. Granados 22015* (FMB, HUA, UPTC), 22016 (COL, FMB, HUA, UPTC, UIS). Casanare. Municipio de Yopal, corregimiento El Morro, vereda Perico, finca La Reserva, 1300–2000 m elev., 17 July 1993, fl, fr, *F. Castro 18718* (FMB, UDBC).

## Discussion

*Microliciacolombiana* is recognized by its strigose nodes with long-stalked glands curled trichomes, and early caducous glands, leaves 3-nerved or 3-plinerved, hypanthium 10-costate, and pale trichomes between the calyx lobes. Other characters, although not unique within the genus but that together help to differentiate *M.colombiana*, are 5-mereous flowers, petals with rounded apex, without seta, glabrous stamens, and polysporangiate anthers. The anthers of species that are polysporangiate have both of their thecae divided into numerous small locules in a way that resembles the structure of a honeycomb (Almeda and Martins 2001), character that can be observed externally without dissecting the anther. The most morphologically similar species is *Microliciabenthamiana* that occurs in Cerro Roraima in Venezuela and Brazil. Both have glabrescent subssesile and 3-nerved leaves, and 10–ribbed hypanthia. In *M.benthamiana*, however, the internodes are shorter, branches and leaves do not have spherical glands and pale trichomes, and the flowers are larger (Table 1). *Microliciacolombiana* also bears some similarity to *M.guanayana*, which occurs on Cerro Guanay in the state of Amazonas, Venezuela. Both have leaves of similar shape and size; however, *Microliciaguanayana* has leaves 1-nerved (vs. 3-nerved in *M.colombiana*), internodes and leaves with strigose indumentum with trichomes > 1 mm long (vs. without setae or only over veins and 0.3 mm long), petals >10 mm long (vs. <8.5 mm long) and setulose and unribbed hypanthia (vs. 10-ribbeb) (Table 1).

**Table 1.** Morphological comparison between *M.colombiana* and related species. Differential characters highlighted in bold. Based in Wurdack (1958, 1973).

Character	* M. colombiana *	* M. guanayana *	* M. benthamiana *
Indumentum on the branch	Frequently puberulous	Strigose - setulose	**Glabrous**
Internode length (mm)	2.4–8.1	3–7	**1.3–2.2**
Petiole length (mm)	0.3–0.7	0.6–0.8	0.8
Leaf shape	Linear to oblong	Linear obovate	**Elliptic**
Leaf length (mm)	4.1–9.9	6–9	4–9
Leaf adaxial surface	Without setae	**Sparse strigose**	Without setae
Leaf abaxial surface	Setae on veins	Sparse strigose	**Without setae**
Venation	3–nerved	**1–nerved**	3–nerved
Pedicel length (mm)	1.2–2	0.5–1.5	**0.8**
Hypanthium length (mm)	3–3.7	3	2–3.5
Hypanthium	10-ribbed	**Smooth**	10-ribbed
Calyx lobe length (mm)	2.1–2.7	3	2–4.6
Indumentum between calyx lobes	Trichomes 0.6–1 mm	?	**Without trichomes**
Petal length (mm)	6.8–8.5	**10–10.5**	**6.8–12**

Regarding the Andean species that grows in Peru and Bolivia, the most affine is *M.sphagnicola* Gleason, nevertheless the latter has flowers 4-merous and glandular marginate connective in outter (antesepalous) stamens.

*Microlicia* is a highly diversified genus in south-central Brazil, with a few disjunct species in the Andes of Bolivia and Peru and the Guiana Shield of Venezuela (Romero 2003a). The most morphologically similar species to *M.colombiana* grow in high areas (1000–2700 m elevation) of the Guiana Shield around 650 km (*M.guanayana*) and 750–1300 km (*M.benthamiana*) away from the northeastern Andes of Colombia (Figure 4A).

This distributional pattern could be explained by long-distance seed dispersal events from the East (southeastern Brazil or Guiana Shield) towards the West (central and northern Andes). In Melastomataceae, there are several different genera with disjunct distribution between the northern Andes and the Guiana Shield, such as in *Boyania* Wurdack (Mendoza 2010) and *Phainantha* Gleason (Ulloa and Neill 2009). Also, there are species with known disjunct distributions between the Guiana Shield and the Andes without records in the lowland of Amazonia and Orinoquia, such as *Marcetiataxifolia* (A. St.-Hil.) DC. (Martins 1989), *Graffenriedaintermedia* Triana and *G.weddelli* Naudin (Almeda et al. 2016), and *Monochaetumbonplandii* (Kunth) Naudin (Alvear and Almeda in press).

The majority of these groups with disjunct distributions have capsular fruits with seeds dispersed by wind. Renner et al. (2001) and Renner (2004) discussed the preponderant role of long-distance dispersal in the evolution of the distribution of Paleotropical Melastomataceae. The small seeds of some of the genera in the Melastomataceae are likely to travel great distances in air currents. The disjunctive distribution of *Microlicia* could possibly be associated with the wind currents in the north of South America that run from East to West (Figure 4B). However, it is possible that the distribution in the center of South America is a combination of stepping-stone route with consecutive short dispersal events.

Another condition is the type of ecosystem, like sandy enclaves in highlands Andes. Successful colonization and establishment are more likely in environments that approximately match the source environment (Cavendar-Bares et al. 2009). The most affine species to *M.colombiana*, grow in what is known as Pantepui (Wurdack 1973), which includes the characteristically flat topped mountain summits of the Guiana highlands, between 1500 and 3000 m a.s.l., encompassing a range of meso‐ to submicrothermic temperature regimes (MAT 18–8 °C), the extensive herbaceous ecosystems are developed on open sandstone surfaces (Huber 2006). These temperature conditions, herbaceous or shrub vegetation and sandy soils occur in some areas along the eastern slope of the eastern Andes in Colombia as the type locality of *M.colombiana*.

Dispersal by birds that transport seeds adhered in mud may also be a possibility to explain the disjunct distribution of *Microlicia* in the northern Andes. However, there is no greater evidence to document this. Bird migrations occur mostly from north-south and not from East to West (Gillespie et al. 2011), and the possibilities of transporting seeds in mud from areas where soils are sandy are unlikely.

## Supplementary Material

XML Treatment for Microlicia colombiana
